# Clinical Studies on Topical Anesthesia in Dentistry: A Bibliometric Analysis

**DOI:** 10.3390/ph18111611

**Published:** 2025-10-24

**Authors:** Helena Carla Gonçalves dos Santos, Iago Torres Cortês de Sousa, Aylla Mesquita Pestana, Arthur Antunes Costa Bezerra, Ana Luiza Martins Lucas, Paulo Antônio Martins-Júnior, Michelle Franz-Montan

**Affiliations:** 1 Department of Biosciences, Piracicaba Dental School, Universidade Estadual de Campinas—UNICAMP, Piracicaba 13414-903, SP, Brazil; gonsahelena@gmail.com (H.C.G.d.S.); iagocortes20@gmail.com (I.T.C.d.S.); pestanaaylla@gmail.com (A.M.P.); arthurantunes32@gmail.com (A.A.C.B.); anamartinsl@outlook.com (A.L.M.L.); 2 Department of Pediatric Dentistry, School of Dentistry, Federal University of Minas Gerais—UFMG, Belo Horizonte 31270-901, MG, Brazil; pauloa-martinsjunior@ufmg.br

**Keywords:** bibliometrics, dental anesthesia, clinical trial, dentistry, local anesthetics, topical anesthesia

## Abstract

Topical anesthesia is widely used in dentistry. However, its clinical effectiveness remains controversial. This study conducted a bibliometric analysis of the most cited clinical dentistry articles on topical anesthesia, identifying the most studied drugs, research trends, leading authors, geographic distribution, funding sources, and study characteristics. A structured search was conducted in the Web of Science using defined descriptors and Boolean operators, with citation comparisons in Scopus and Google Scholar. The search, conducted without language or publication year restrictions, extended until October 2023. Out of 4892 articles, the 30 most cited were selected, collectively accumulating 953 citations between 1992 and 2017. Most highly cited and funded studies originated from the United States. Journals focusing on periodontics, general dentistry, and oral surgery published the majority of these articles, with lidocaine being the most studied anesthetic. Despite their widespread use, topical anesthetics have garnered relatively few citations. Our findings suggest a research preference for studies with immediate clinical impact over those focusing on pain management, particularly in the field of topical dental anesthesia. This highlights the need for further research and progress in this important field.

## 1. Introduction

Fear and anxiety commonly associated with needles and injections can prevent patients from seeking regular dental treatment [1]. The pain associated with injections, particularly in areas such as the hard palate and or during regional block and intra-ligamentary techniques, can be quite intense [2,3,4,5,6]. When associated with anxiety, patients often report higher levels of pain [7]. To provide greater comfort to patients during local anesthesia, dentists commonly use topical anesthetics.

Topical anesthetics are semi-solid formulations used in various healthcare fields, including dentistry. They differ from injectable “local anesthetics”, which consist of injectable liquid formulations used to promote local or regional anesthesia during dental procedures. In contrast, topical anesthetics in dentistry act on the oral mucosa, aimed at reducing discomfort at the injection site of local anesthetic solutions. Furthermore, these not only reduce pain during puncture but also alleviate discomfort caused by periodontal treatment, mucosal lesions, biopsies, restorative procedures, placement of orthodontic bands, or even simple tooth extractions [8,9,10,11].

Just like injectable local anesthetic solutions, topical anesthetics in semi-solid formulations contain amide or ester-type local anesthetics. The most commonly used in medicine and dentistry are lidocaine and prilocaine (amides), as well as benzocaine, tetracaine hydrochloride, and butamben (esters). Some formulations also include dyclonine hydrochloride, a compound that does not belong to either the amide or ester class, and is instead classified as an atypical (ketone-type) local anesthetic due to its distinct chemical structure [12,13,14,15]. Combination formulations are also frequently used, including mixtures of benzocaine, butamben, and tetracaine, or the eutectic mixture of lidocaine and prilocaine (EMLA^®^), originally developed for cutaneous application but increasingly studied for dental use on the oral mucosa [14,16].

Topical anesthetics are applied directly to the oral mucosa but still require tissue permeation to reach their site of action and produce anesthesia. Unlike injectable anesthetics, which are delivered via needle directly to the neuronal target, topical formulations must overcome the epithelial barrier, which can limit their effectiveness. Moreover, factors such as the integrity of the epithelium and the continuous presence of saliva, constantly washing the application site, further compromise drug retention and absorption. As a result, the clinical effectiveness of topical anesthetics remains under investigation and is still considered controversial [16].

Commercially available formulations include gels, ointments, solutions, and adhesive patches [1,16,17,18]. When topically applied to the oral mucosa, they typically produce anesthesia in the superficial 2–3 mm of tissue [19]. However, the effectiveness of existing formulations varies in the literature, prompting research into the development of new pharmaceutical formulations to enhance efficacy and minimize toxicity [20,21,22,23,24,25,26,27,28].

Historically, topical anesthetics emerged in the late 19th century, beginning with cocaine [29,30]. Cocaine, an alkaloid derived from the coca leaf, found in Peru and Bolivia, was isolated by chemist Niemann in 1859 and was later introduced as an anesthetic by Koller in 1884 [29]. However, the use of cocaine quickly became limited due to its toxicity and the risk of acute dependence [1]. The development of safer local anesthetics was relatively slow. After 20 years, Alfred Einhorn developed procaine, a synthetic and safer local anesthetic, in 1904. Nearly 30 years later, tetracaine was discovered in 1932, followed by the first non-ester, widely considered the safest and gold-standard anesthetic, lidocaine, in 1943, which was introduced in the market as Xylocaine in 1949 [31]. Today, benzocaine and lidocaine are the most commonly used local anesthetics in dentistry, representing the ester and amide types, respectively [1].

Considering this context, we conducted a bibliometric analysis to identify the most cited clinical studies on topical anesthesia in dentistry. The analysis encompasses the most researched drugs, active research groups, authors, and institutions in terms of citations, the global distribution of studies, journals with the highest publications, funding involvement, publication period, evolution of topics on anesthesia over the years, as well as specific characteristics of studies related to the effectiveness of the tested anesthetics. A bibliometric review of existing studies on topical anesthesia is an important task in this context. Analyzing the characteristics of published articles can provide insights into relevant research questions, identify key researchers, and map the geographic distribution of research [32]. This, in turn, can guide future investigations in the field.

## 2. Results

**Figure 1:**
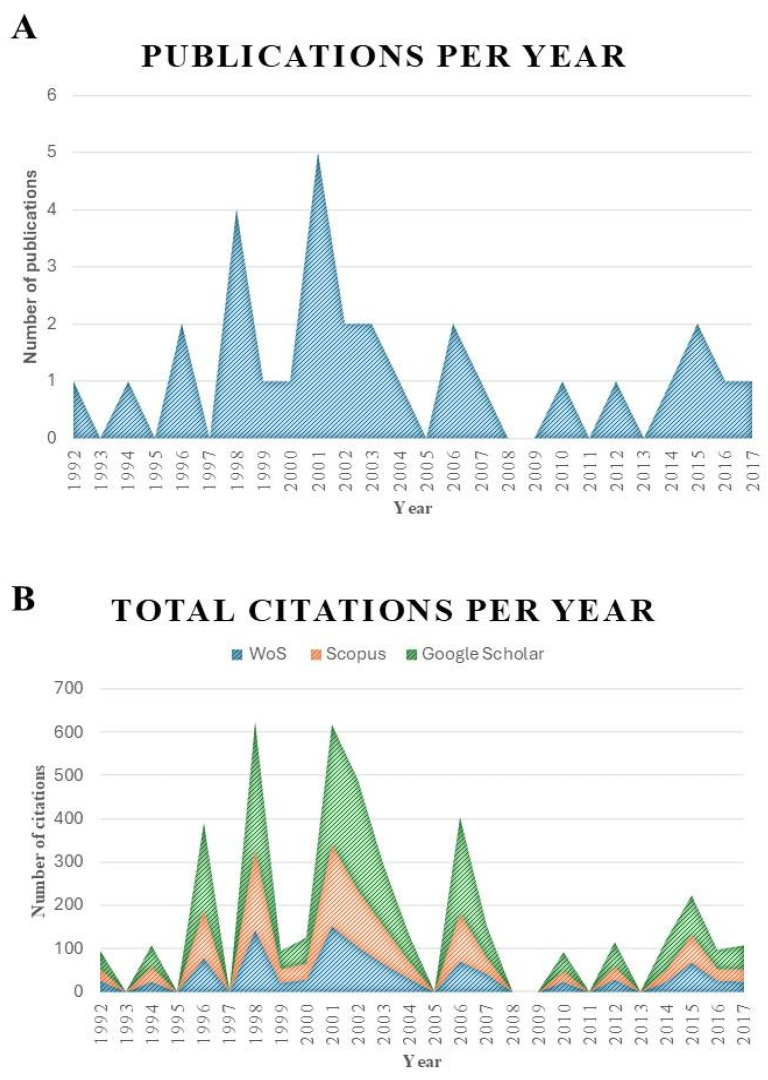
Number of publications and citations per year based on the Web of Science, Scopus, and Google Scholar platforms. (**A**) Total publications per year. (**B**) Total citations per year.

A search key yielded 4892 articles from which the 30 most cited articles were selected. Table 1 presents the titles of the selected articles, authors, publication year, and number of citations (including self-citations). The publications listed in this work cover a period of 25 years, with numbers of citations ranging from 21 to 70, according to data provided by WoS-CC. Citation counts were reported in other databases (Table 1). Together, the 30 most cited articles accumulate 974 citations in WoS-CC, 1215 in Scopus, and 2093 in Google Scholar. Figure 1 shows the number of articles published per year and the number of citations per year based on the thirty selected articles.

The clinical studies varied in their objectives, tested substances, and findings. Table 2 summarizes some parameters and methodological criteria, as well as the central results of each study. The most cited article in the three databases searched is about the effectiveness of an orthodontic wax containing benzocaine released over time in a controlled manner in reducing pain related to trauma resulting from the friction of orthodontic appliances on the oral mucosa [33]. The article with the highest citation density, according to WoS-CC and Scopus, describes a liposomal lidocaine for topical use on the oral mucosa. It includes results from the development stage to a clinical trial evaluating anesthetic efficacy in volunteers [23]. However, according to Google Scholar, the article with the highest citation density is about the comparison between natural clove and benzocaine as a topical anesthetic in reducing pain during puncture [34].

**Figure 2:**
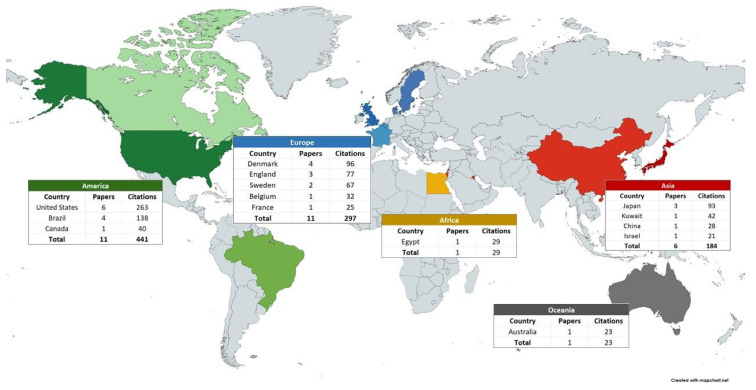
Distribution of articles by country, based on the corresponding authors’ affiliations. Each color denotes a continent, and darker shades indicate countries with a higher number of articles and citations among the 30 most-cited publications. For countries with only one citation, there is no difference in color shade.

**Figure 3:**
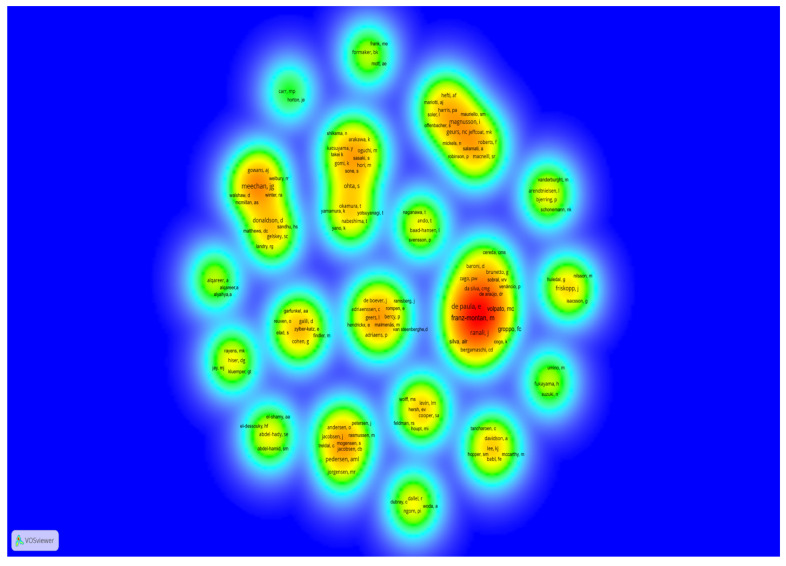
Co-authorship map showing dominant research groups. Larger circles indicate greater prominence of groups in Brazil, the United States, England, Japan, Canada, Belgium, and Denmark. Each point on the density map is colored according to the concentration of items in that region: the higher the number and weight of nearby items, the closer the color is to red; conversely, fewer and lighter items result in colors closer to blue.

The most cited papers originated from countries in the Americas (36.7%) and Europe (36.7%). The geographical distribution of the corresponding authors of the publications, the main institutions, journals, funding, as well as characteristics of the articles, such as number of authors and year of publication, are presented in Figure 2 and Table 3. Based on the co-authorship analysis (Figure 3), research groups in Brazil, the United States, England, Japan, Canada, Belgium, and Denmark dominate the field. Additionally, Table 4 presents the bibliometric characteristics of the 10 most cited authors. In total, 133 authors were listed in the 30 articles.

**Figure 4:**
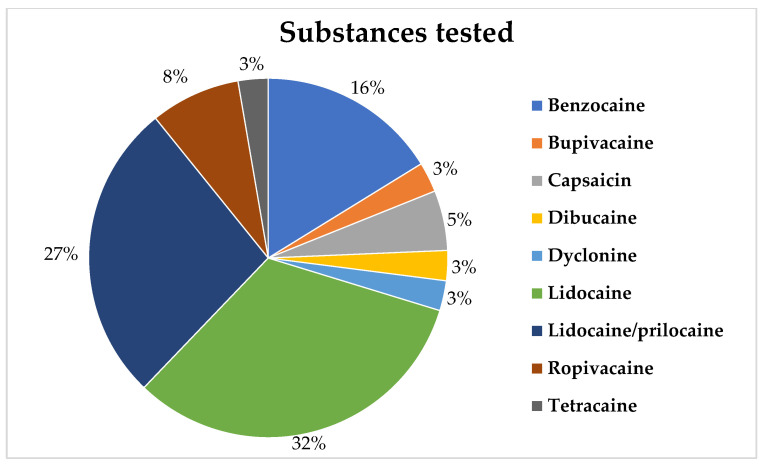
Local anesthetics most frequently investigated in the cited articles.

Among the studies, lidocaine was the most researched drug (42.9%), with prilocaine (26.5%) and benzocaine (14.3%) following closely behind (Figure 4). It is worth mentioning that several studies examined combined anesthetics in formulations, such as EMLA^®^ and Oraqix^®^ (both containing a eutectic mixture of lidocaine and prilocaine). Among the combinations assessed in the 30 articles, EMLA^®^ was featured in 7 studies, totaling 23.3% of the studies, followed by Oraqix^®^, which was evaluated in 4 studies, totaling 13.3%.

**Figure 5:**
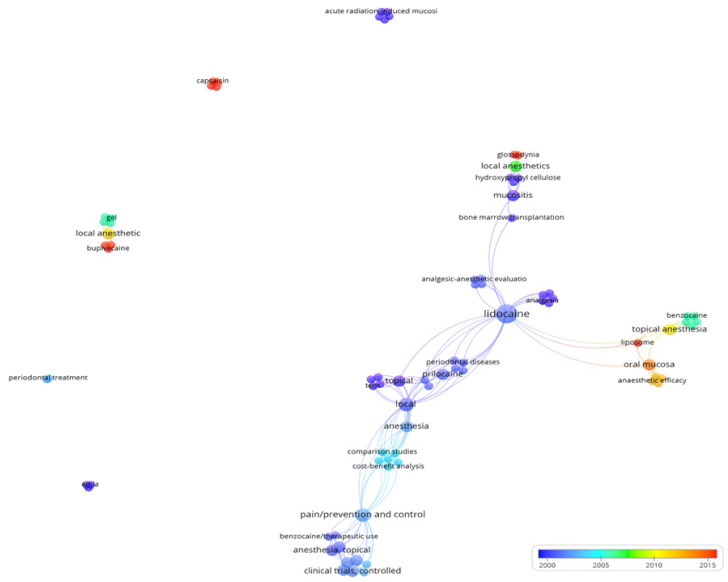
Overlay map showing the 83 identified keywords over the years of publication.

**Figure 6:**
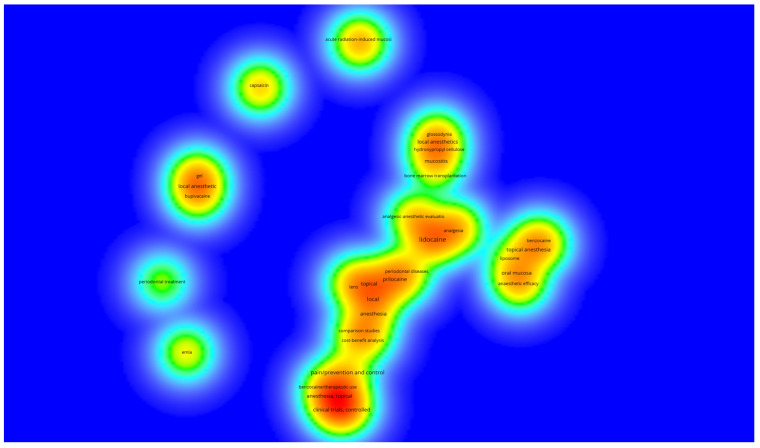
Density map of the 83 identified keywords. Warmer colors (closer to red) indicate a higher number of articles using that keyword, while fewer articles result in colors closer to blue.

Finally, in the keyword analysis, a total of 83 keywords were identified across the studies. Notably, lidocaine emerged as a frequently cited anesthetic, often linked with other medications like prilocaine. Terms such as periodontal disease, analgesia, mucositis, and liposomes have also gained attention in recent research (Figure 5). Keywords including ‘lidocaine’, ‘local anesthesia’, ‘clinical trials’, ‘prilocaine’, ‘liposome’, ‘mucositis’, and ‘benzocaine’ were highlighted for their density, indicating frequent occurrence across the articles (Figure 6).

**Table 1:** Thirty most cited articles on topical anesthesia in Dentistry according to Web of Science Core Collection and comparison with other databases.

Rank	Article Title	Corresponding Author	Country (According to the Address of the Corresponding Author)	Year of Publication	Total Citations (Citation Density *)		
					Web of Science—Core Collection	Scopus	Google Scholar
1	Efficacy of a wax containing benzocaine in the relief of oral mucosal pain caused by orthodontic appliances [33]	Kluemper, GT.	USA	2002	70 (3.5)	86 (4.3)	178 (8.9)
2	The effects of topical anesthesia on oral burning in burning mouth syndrome [35]	Formaker, BK.	USA	1998	60 (2.5)	82 (3.42)	115 (4.8)
3	Analgesic efficacy and safety of an intraoral lidocaine patch [9]	Hersh, EV.	USA	1996	48 (1.85)	68 (2.62)	121 (4.66)
4	Liposomal lidocaine gel for topical use at the oral mucosa: characterization, in vitro assays and in vivo anesthetic efficacy in humans [23]	Franz-Montan, M.	Brazil	2015	46 (6.57)	44 (6.29)	68 (9.72)
5	The effect of clove and benzocaine versus placebo as topical anesthetics [34]	Alqareer, A.	Kuwait	2006	42 (2.63)	73 (4.56)	171 (10.67)
6	The anesthetic onset and duration of a new lidocaine/prilocaine gel intra-pocket anesthetic (Oraqix^®^ for periodontal scaling/root planing [36]	Friskopp, J.	Switzerland	2001	42 (2.0)	47 (2.24)	71 (3.38)
7	Liposome-encapsulated ropivacaine for topical anesthesia of human oral mucosa [27]	Groppo, FC.	Brazil	2007	41 (2.73)	37 (2.47)	73 (4.87)
8	A placebo-controlled multi-centred evaluation of an anaesthetic gel (Oraqix^®^) for periodontal therapy [21]	Donaldson, D.	Canada	2003	40 (2.11)	53 (2.79)	82 (4.32)
9	Intrapocket anesthesia for scaling and root planing: Results of a double-blind multicenter trial using lidocaine prilocaine dental gel [37]	Jeffcoat, MK.	USA	2001	37 (1.76)	45 (2.14)	78 (3.72)
10	Mucosa-adhesive water-soluble polymer film for treatment of acute radiation-induced oral mucositis [20]	Oguchi, M.	Japan	1998	35 (1.46)	46 (1.92)	66 (2.75)
11	Comparison of topical anesthesia of 20% benzocaine and 60% lidocaine gel [38]	Fukayama, H.	Japan	2002	34 (1.7)	45 (2.25)	80 (4.0)
12	Patient evaluation of a novel non-injectable anesthetic gel: A multicenter crossover study comparing the gel to infiltration anesthesia during scaling and root planing [2]	van Steenberghe, D.	Belgium	2004	32 (1.78)	39 (2.17)	63 (2.5)
13	Formulation of an antispasmodic drug as a topical local anesthetic [39]	Abdel-Hamid, SM.	Egypt	2006	29 (1.81)	36 (2.25)	52 (3.25)
14	A comparison of topical anaesthesia and electronic nerve stimulation for reducing the pain of intra-oral injections [40]	Meechan, JG.	England	1996	29 (1.16)	42 (1.62)	82 (3.16)
15	Liposomal delivery system for topical anaesthesia of the palatal mucosa [26]	Franz-Montan, M.	Brazil	2012	28 (2.8)	30 (3.0)	56 (5.6)
16	The efficacy of EMLA^®^ and 5% lignocaine gel for anaesthesia of human gingival mucosa [41]	McMillan, AS.	China	2000	28 (1.27)	36 (1.64)	61 (2.77)
17	Onset and duration of hypoalgesia of lidocaine spray applied to oral mucosa—A dose–response study [42]	Van der Burght, M	Denmark	1992	26 (0.87)	27 (0.9)	40 (1.33)
18	Effect of a local anesthetic lozenge in relief of symptoms in burning mouth syndrome [22]	Treldal, C.	Denmark	2016	25 (4.17)	28 (4.67)	44 (7.33)
19	Intrapocket anesthesia for scaling and root planing in pain-sensitive patients [43]	Magnusson, I.	USA	2003	25 (1.32)	33 (1.74)	62 (3.26)
20	A human oral capsaicin pain model to assess topical anesthetic-analgesic drugs [44]	Dallel, R.	France	2001	25 (1.19)	29 (1.38)	41 (1.95)
21	Plasma levels of lidocaine and prilocaine after application of Oraqix^®^, a new intrapocket anesthetic, in patients with advanced periodontitis [45]	Friskopp, J.	Switzerland	2001	25 (1.19)	36 (1.72)	43 (2.05)
22	Analgesic effect of topical oral capsaicin gel in burning mouth syndrome [46]	Jørgensen, MR	Denmark	2017	24 (4.0)	28 (5.6)	56 (9.33)
23	Oral mucosal adhesive film containing local anesthetics: In vitro and clinical evaluation [47]	Yamamura, K.	Japan	1998	24 (1.0)	25 (1.04)	49 (2.04)
24	The use of patient-controlled transcutaneous electronic nerve stimulation (TENS) to decrease the discomfort of regional anaesthesia in dentistry: Randomized controlled clinical trial [48]	Meechan, JG.	England	1998	24 (1.0)	34 (1.42)	63 (2.63)
25	The intraoral use of EMLA cream in children—A clinical investigation [49]	Meechan, JG.	England	1994	24 (0.86)	34 (1.22)	50 (1.79)
26	Topical Lidocaine to Improve Oral Intake in Children With Painful Infectious Mouth Ulcers: A Blinded, Randomized, Placebo-Controlled Trial [50]	Hopper, SM.	Australia	2014	23 (2.56)	26 (3.25)	72 (8.0)
27	Liposome-encapsulated ropivacaine for intraoral topical anesthesia [25]	Franz-Montan, M.	Brazil	2010	23 (1.92)	25 (2.08)	43 (3.58)
28	Clinical evaluation and comparison of 2 topical anesthetics for pain caused by needle sticks and scaling and root planing [51]	Carr, MP.	USA	2001	23 (1.1)	30 (1.43)	46 (2.19)
29	Influence of topical application of capsaicin, menthol and local anesthetics on intraoral somatosensory sensitivity in healthy subjects: temporal and spatial aspects [52]	Naganawa, T.	Denmark	2015	21 (3.0)	19 (2.72)	25 (3.57)
30	Systemic absorption of lidocaine after topical application for the treatment of oral mucositis in bone marrow transplantation patients [53]	Elad, S	Israel	1999	21 (0.91)	32 (1.39)	42 (1.83)

* The values in parentheses correspond to the citation density, calculated based on the following equation: number of citations in the database/(2022 − year of publication).

**Table 2:** Methodological characteristics of the studies cited, and main results found. The order of articles was defined based on the number of citations (from the most cited article to the least cited).

Rank	Author (Year)	Main Objective	Drugs Used	Type of Formulations	Mouth Region	Time of Application	No. of Volunteers	Age	Main Results
1	Kluemper et al., (2002) [33]	Determine the effectiveness of an orthodontic wax containing benzocaine, which is released over time in a controlled manner, for reducing pain related to trauma resulting from friction of orthodontic appliances on the oral mucosa.	Benzocaine (20%)	Wax	Oral mucosa	The participants used the wax during the 53 h pain assessment.	70	Adults and children, with no information about the age range.	The wax was significantly effective in reducing discomfort in the mucosa associated with orthodontic appliances.
2	Formaker et al., (1998) [35]	Test the effectiveness of a topical anesthetic in patients with oral burning and taste alterations.	Dyclonine HCl 1%	Solution	Oral mucosa	2 min	33	35–83 years	Dyclonine promoted an increase, reduction, and absence of change in patients with burning mouth syndrome. Regarding taste, dyclonine led to a reduction in dysgeusia.
3	Hersh et al., 1996 [9]	Test the effectiveness of intraoral lidocaine patches in the pain experience during the puncture of a 25 G needle.	Lidocaine 10% and 20%	patches	2 mm apical to the mucogingival junction in both the maxillary and mandibular premolar region.	2 min	101	18–65 years	Lidocaine patches at both concentrations were effective and safe in reducing needle insertion pain.
4	Franz-Montan et al., 2015 [23]	Characterize liposomal formulations of lidocaine for topical use on the oral mucosa and compare their in vitro permeation and in vivo anesthetic efficacy.	5% lidocaine encapsulated in liposomes, 2.5% lidocaine encapsulated in liposomes, 5% lidocaine ointment, eutectic mixture of lidocaine and prilocaine 2.5% (EMLA)^®^	Gel	Palatal mucosa of the upper canines.	2 min	40	18–29 years	5% lidocaine encapsulated in liposomes and EMLA showed the best in vitro results, as well as the best topical anesthetic efficacy in vivo.
5	Alqareer et al., 2006 [34]	Verify if natural clove can replace benzocaine as a topical anesthetic in reducing pain during puncture.	Clove gel (prepared with commercially available clove powder mixed with glycerin), 20% benzocaine, and placebo.	Gel	Buccal mucosa of the upper canines.	4 min	73	19–25 years	Both clove gel and benzocaine showed lower pain scores compared to the placebo, with no statistical difference between the two active agents.
6	Friskopp et al., 2001 [36]	Determine the onset and duration of the anesthetic gel (Oraqix^®^) in patients with periodontitis during scaling and root planing procedures.	Lidocaine (25 mg/g) + Prilocaine (25 mg/g) (Oraqix^®^)	Gel	Periodontal pocket	30 s, 2 min and 5 min.	30	18–60 years	Oraqix^®^ provides anesthesia 30 s after application and has a duration of effect close to 17–20 min.
7	Franz-Montan et al., 2007 [27]	Evaluate the effectiveness of ropivacaine encapsulated in liposomes for topical anesthesia.	1% ropivacaine encapsulated in liposomes, 1% plain ropivacaine, a mixture of 2.5% lidocaine and 2.5% prilocaine (EMLA^®^), and 20% benzocaine	Gel	Buccal fold of the upper-right canine	2 min	30	18–26 years	The 1% ropivacaine gel encapsulated in liposomes was equivalent to EMLA in reducing pain during needle insertion and for the duration of anesthesia in soft tissue. None of the topical anesthetics was effective in inducing pulp anesthesia.
8	Donaldson et al., 2003 [21]	The ability of a thermosetting gel to produce analgesia in periodontal pockets during scaling and root planing.	Lidocaine (25 mg/g) + Prilocaine (25 mg/g) (Oraqix^®^)	Gel	Periodontal pocket	30 s–2 min	130	18+ years	The 5% anesthetic gel was statistically more effective than the placebo in reducing pain during periodontal debridement surgery.
9	Jeffcoat et al., 2001 [37]	Evaluate the effectiveness of a new anesthetic gel for periodontal pocket anesthesia.	Lidocaine 25 mg/g with prilocaine 25 mg/g	Gel	Periodontal pocket	30 s–2 min	122	18+ years	The gel showed a significant reduction in pain compared to the placebo, indicating its potential effectiveness/utility during scaling and root planing procedures.
10	Oguchi et al., 1998 [20]	Examine the usefulness and safety of a water-soluble and mucoadhesive polymeric film containing anesthetics and antibiotics for the treatment of radiation-induced acute oral mucositis.	Hydroxypropyl cellulose (600 mg), Tetracaine Hydrochloride (5 mg), Ofloxacin (5 mg), Miconazole (5 mg), Guaiazulene (0.6 mg), Triacetin (24 mg), ethyl alcohol (100 mL)	Adhesive	Oral mucosa	1 h	52	34–87 years	It proved to be useful for relieving pain resulting from acute radiation-induced oral mucositis, maintaining good oral nutrition, and preventing secondary oral infections, without inducing adverse reactions.
11	Fukayama et al., 2002 [38]	Determine the effectiveness of 2 commonly used topical anesthetics in dentistry.	Benzocaine 20% and lidocaine 60%	Gel	Alveolar mucosa of the apices of the upper incisors.	20 min	20	23–34 years	The 20% benzocaine showed no change in pain perception when compared to the placebo. However, the 60% lidocaine significantly reduced this perception.
12	van Steenberghe et al., 2004 [2]	Evaluate the preferences of volunteers for the use of non-injectable or injectable anesthetics during scaling and/or root planing.	Lidocaine 25 mg/g and prilocaine 25 mg/g versus injection anesthesia (lidocaine 2% with adrenaline)	Gel and solution	Periodontal pocket of upper and lower quadrants.	30 s	170	25–72 years	The majority of patients (70%) preferred topical anesthesia. 96% of patients reported satisfactory anesthesia with the injection, and 80% with the gel. Post-procedure issues, such as numbness and pain, were lower when the gel was used compared to the injection.
13	Abdel-Hamid et al., 2006 [39]	Formulate mebeverine HCl into a gel that could be used locally in the treatment of various oral painful conditions.	Mebeverine HCl and Lidocaine HCl 20%.	Gel	Oral Cavity	3 to 4 times a day for 2 days.	25	18–65 years	The gel formulation has shown a better pain reduction efficiency and longer duration than Lidocaine HCl gel^®^.
14	Meechan et al., 1996 [40]	Investigate the effectiveness of using EMLA and TENS in relieving pain from intraoral injections prior to extractions of maxillary teeth.	EMLA^®^, TENS^®^, and placebo, before the injection of 2% lidocaine.	Cream in patches; TENS (electrodes);	Palatal mucosa	EMLA and placebo: 5 min; TENS: 2 min	100	18+ years	EMLA reduced the pain of the palatal injection compared to the placebo group. TENS did not differ from the placebo group. Therefore, EMLA may be useful for reducing the pain of injection in the palatal region.
15	Franz-Montan et al., 2012 [26]	Evaluate the efficiency of ropivacaine encapsulated in liposomes at different concentrations for topical anesthesia in the palate.	Ropivacaine 2% encapsulated in liposomes; ropivacaine 1% encapsulated in liposomes; placebo liposomes; and lidocaine 2.5% with prilocaine 2.5% (EMLA^®^)	Gel	Palatal mucosa of the upper canines.	5 min	40	19–29 years	The ropivacaine formulations encapsulated in liposomes were not effective in reducing puncture pain, with no differences compared to the placebo. EMLA was effective in reducing pain during puncture. However, none of the formulations were effective in reducing pain from local anesthetic injection when compared to the placebo.
16	McMillan et al., 2000 [41]	Compare the analgesic effect of a mixture of 2.5% lidocaine and 2.5% prilocaine (EMLA^®^) with 5% lidocaine gel alone for minor gum operations.	EMLA^®^ (Lidocaine 2.5% and prilocaine 2.5%)	Gel	Palatal mucosa	10 min	10	20–21 years	EMLA showed a longer duration of anesthesia and a better area under the pressure-pain threshold curve than lidocaine alone.
17	Schønemann et al., 1992 [42]	Determine the ideal time interval for dental procedures involving pain after the application of lidocaine spray. Pain was tested through stimulation with argon laser.	Lidocaine (30 and 60 mg)	Spray	Lower lip mucosa	5 s	24	Lidocaine 30 mg group: average age of 29.5 years. Lidocaine 60 mg group: average age of 34 years.	There was an increase in the pain threshold for both dosages, with no difference between the groups. Local oral application did not produce complete analgesia. Maximum hypoalgesia occurred between 4 and 5 min, with a duration of up to 14 min. However, the ideal time interval for procedures was between 3-8 min.
18	Treldal et al., 2016 [22]	Evaluate the effect of a bupivacaine lozenge on oral mucosa pain, xerostomia, and taste alterations in patients with Burning Mouth Syndrome.	Bupivacaine	Lozenge	Oral cavity	Use until the tablet is completely dissolved	18	39–71 years	Bupivacaine lozenge significantly reduced burning oral pain, increased the sensation of taste disturbances, and had no impact on xerostomia.
19	Magnusson et al., 2003 [43]	To evaluate the anesthetic effect of the mixture of lidocaine and prilocaine in pain-sensitive patients using a visual analogue scale (VAS) and a verbal rating scale (VRS).	Lidocaine 2.5 mg/g with prilocaine 2.5 mg/g (Oraqix^®^)	Gel	Periodontal pocket	30–45 s	85	21–77 years	The overall median VAS pain score was 11 mm in the treated group and 27 mm in the placebo group. No pain or only mild pain was reported by 70% in the anesthetic group and by 48% in the placebo group. Two patients in the treated group and 7 patients in the placebo group required additional anesthesia.
20	Ngom et al., 2001 [44]	To evaluate the reliability of the oral pain model of response to repeated applications of capsaicin	0.25, 0.5 and 1% lidocaine	Mouthwash (30 mL)	Oral mucosa (tongue)	3 min	29	21–26 years	The capsaicin oral pain model has good reliability and sensitivity and allows safe evaluation of candidate topical analgesics and anesthetics.
21	Friskopp et al., 2001 [45]	To describe the plasma profiles of lidocaine and prilocaine after a single dose of Oraqix^®^ for patients with advanced periodontitis.	Lidocaine/prilocaine (Oraqix^®^) (0.9 to 3.5 g)	Gel	Periodontal pocket	Oraqix^®^ was applied in the pockets around all the teeth. Directly there after all the pockets were probed, teeth were subjected to SRP, and the mouth was then rinsed out with a glass of water	10	38–56 years	Maximum plasma concentrations of lidocaine (99–266 ng/mL) and prilocaine (46–118 ng/mL) occurred 20–40 min after the start of application (below toxicity levels in the Central Nervous System). Therefore, there is a large margin of safety when it comes to applying up to 3.5 g of Oraqix^®^ in periodontal pockets.
22	Jørgensen et al., 2017 [46]	To investigate the efficacy of repeated topical application of capsaicin oral gel at two different concentrations to alleviate burning/stinging sensations in patients with burning mouth syndrome (BMS)	0.01% and 0.025% capsaicin	Gel	Dorsum of the tongue	Three times daily for 14 days, followed by 14 days wash-out period, and finally treatment with the other concentration of oral gel three times daily for 14 days.	22	+18 years	Topical capsaicin may be an alternative for short-term treatment of BMS. However, more studies are needed to investigate, especially the gastrointestinal side effects that may limit its long-term use.
23	Yamamura et al., 1998 [47]	To evaluate the effectiveness of an adhesive film for oral mucosa containing dibucaine (DC) in relieving pain caused by oral erosion.	Dibucaine	Adhesive film containing two concentrations: 0.113 and 0.225 mg/cm^2^	Left buccal mucosa	No information	23	29–66 years	The film showed good adhesion to the mucosa. The onset of the anesthetic effect was less than 5 min for both concentrations tested. The duration of anesthesia was 2.2 ± 0.21 h for the 0.113 mg/cm^2^ group and 4.3 ± 0.25 h for the 0.225 mg/cm^2^ group.
24	Meechan et al., 1998 [48]	Compare the use of topical anesthesia and transcutaneous electrical stimulation (TENS) as a means of reducing discomfort from lower tooth block injections	20% Benzocaine, TENS and no pretreatment	Benzocaine: Gel; TENS: electrodes.	Oral mucosa	2 min	100	+18 years	The discomfort of the long buccal injection did not differ between the three groups of patients. For lower tooth block anesthesia, the injection discomfort after TENS was lower than that in other groups. Topical anesthetic did not produce a significant change in injection discomfort compared to no pretreatment.
25	Meechan et al., 1994 [49]	Efficacy of EMLA^®^ in Local Topical Anesthesia on the Palate Compared to Other Local Anesthetics	EMLA^®^ and lidocaine 5%.	Gel	Fold of the oral mucosa	5 min	20	6–15 years	There is no advantage to be gained from using EMLA compared to conventional intraoral topical anesthetics when placed in the buccal fold in children.
26	Hopper et al., 2014 [50]	To establish the effectiveness of viscous 2% lidocaine in increasing oral intake in children with oral infectious conditions compared to placebo.	2% lidocaine	Viscous lidocaine	Oral mucosa	60 min	110	6 months–8 years	Viscous lidocaine is not superior to a flavored gel placebo in improving oral intake in children with painful infectious mouth ulcers.
27	Franz-Montan et al., 2010 [25]	To evaluate the efficacy of 2% ropivacaine encapsulated in liposomes in topical anesthesia and its influence on pulp response.	2% ropivacaine encapsulated in liposomes; 20% benzocaine, placebo encapsulated in liposomes; placebo liposomal gel; and placebo gel.	Gel	Vestibular groove of the lateral region of the maxilla-incisive region (bilateral)	30 min	40	18–43 years	Ropivacaine gel 2% and benzocaine 20% showed a lower response to needle insertion pain and long anesthesia in soft tissues, when compared to placebo, with no differences between the first two formulations. However, none were able to induce pulpal anesthesia.
28	Carr et al., 2001 [51]	To evaluate the effectiveness of the transoral lidocaine delivery system and compare it with a benzocaine gel preparation in reducing pain for needle stick and scaling and root planing.	60% Lidocaine and 20% benzocaine	Adhesive and gel	Maxillary and mandibular molar-bicuspid areas	15 min (lidocaine) 30 s (gel benzocaine)	40	21–70 years	The transoral lidocaine delivery system is more effective than benzocaine gel in topical pain suppression for needle sticks and root planing in both arches.
29	Naganawa et al., 2015 [52]	To investigate temporal and spatial aspects of somatosensory changes after topical application of capsaicin, menthol and local anesthetics to the gums using intraoral palpometers and thermal devices.	EMLA^®^ (lidocaine with prilocaine 5%)	Cream	Gum in the region of the upper first premolar	15 min	16	Average age = 29 years	Topical application of capsaicin, but not menthol, increased thermal, but not mechanical, sensitivity at the application site and adjacent test sites, indicating thermal hyperalgesia in the primary zone, and for the first time also in the secondary gingival zone.
30	Elad et al., 1999 [53]	To evaluate the absorption of lidocaine by the oral mucosa after its topical administration in the symptomatic treatment of oral mucositis induced by bone marrow transplantation.	2% lidocaine	Mouthwash	Oral mucosa	1 min	10	6–48 years	Lidocaine prescribed as an anesthetic mouthwash in patients with bone marrow transplant and oral mucositis, results in less systemic absorption of the drug.

**Table 3:** Institutional characteristics, journals, funding, authors, and publication years of the articles cited more than twice among the 30 most cited.

Characteristics		N	Citations
**Main Institutions**			
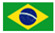	Universidade Estadual de Campinas	4	138
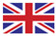	University of Newcastle	3	77
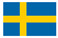	Public Dental Services of Sweden	2	67
**Main journals**			
Journal of Periodontology		4	117
Journal of Clinical Periodontology		3	107
Journal of Dentistry		2	66
Oral Surgery, Oral Medicine, Oral Pathology, Oral Radiology, and Endodontology		2	57
British Journal of Oral and Maxillofacial Surgery		2	56
**Funding/continent**			
***America***			
Funded		8	282
Not Funded		3	159
***Europe***			
Funded		4	102
Not Funded		7	195
***Asia***			
Funded		2	63
Not Funded		4	121
***Africa***			
Funded		0	-
Not Funded		1	29
***Oceania***			
Funded		0	-
Not Funded		1	23
**Number of Authors/Articles**		**N**	
1– 4		16	
5–8		10	
>8		4	
**Publication Time Range**		**N**	
1992–1996		4	
1997–2001		11	
2002–2006		7	
2007–2011		2	
2012–2017		6	

**Table 4:** Bibliometric indicators of the 10 authors with the highest number of articles among the 30 most cited.

Authors		Number of Articles Among the 30 Most Cited	Total Citations in the 30 Most Cited Articles	Authorship Position	Number of WoS-CC Articles	Number of WoS-CC Citations	H-Index
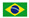	Franz-Montan M.	4	138	First author on all	65	1413	23
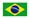	Groppo FC.	4	138	Co-author: 2 Last author: 2	137	1781	22
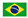	Volpato MC.	4	138	Co-author: 2 Last author: 2	66	835	18
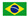	de Paula E.	4	138	Co-author on all	220	5248	39
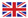	Meechan JG. *	4	105	First author: 3 Last author: 1	164	2386	27
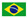	Ranali J *	3	92	Co-author on all	37	411	12
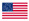	Kluemper GT *	1	70	First author: 1	27	656	13
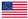	Jay MJ	1	70	Last author: 1	138	3602	33
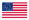	Rayens MK *	1	70	Co-author: 1	295	4810	36
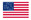	Hiser DG *	1	70	Co-author: 1	2	77	2

* This is an algorithmically generated author record. WoS-CC: Web of Science Core Collection. Data regarding the number of articles and citations in WoS-CC and the H index were updated in June 2024.

## 3. Discussion

In this review, we compiled the 30 most cited clinical studies on topical anesthetics in Dentistry, presenting information on citation counts, main research areas, tested anesthetic agents, key findings, and bibliometric indices, including leading authors and research groups, funding sources, journals, geographic distribution, and keyword evolution. We opted to select articles with at least 20 citations, resulting in 34 articles that met this criterion. Given that there is no established rule for the minimum or maximum number of articles to be included in a bibliometric study, we decided to include the 30 most cited articles. In broader fields of dentistry, studies often include a high number of papers, such as 100 [54,55], 300 [56,57], or even more than 3000 [58]. However, specific topics typically include fewer papers, such as the top 30 [59] or top 50 [60].

Interestingly, topical dental anesthesia exhibits a much lower citation rate compared to other dental fields, such as Cariology [54], Endodontics [60], Periodontics [61,62], Dental Materials and Restorative Dentistry research [63], among others. In the present bibliometric analysis, the most cited article achieved 70 citations in the WoS database. In contrast, some articles in these other areas have exceeded 3000 citations. For example, a study in periodontics by Socransky received 3247 citations [64], and a study in implantology by Adell et al. received 3155 citations [65], according to WoS data.

A bibliometric study identified the 100 most cited articles in Dentistry [66], at the time of its publication, included articles ranging from 326 to 2050 citations (ISI Web of Knowledge). Among the top 10 most cited, notable works are in the areas of restorative Dentistry/dental materials [67,68], implantology [65,69,70], periodontology [64,71,72], and surgery/pathology [73,74]. Many of these articles are considered classics in dental literature and are relatively older. In Dentistry, research in public health, pediatric dentistry, restorative and adhesive dentistry, as well as implantology, has been among the most researched topics in recent decades [58]. None of the listed articles is related to dental anesthesia.

Although older publications have a greater opportunity to accumulate citations over the years [66], this trend was not observed in the present study. The oldest study among the top 30 was published in 1992 (Rank 17) and accumulated 26 citations [42], whereas the most recent study, published 25 years later in 2017, received a similar number of citations (24 citations, Rank 22) [46]. This can be attributed to the latter’s theme, which involved a capsaicin pain model.

Regarding the drugs used, the studies varied in their investigations. Lidocaine was observed to be the most studied local anesthetic, followed by prilocaine and benzocaine. However, other anesthetics were also tested, including ropivacaine, evaluated for its efficacy in reducing needle pain [25,26], bupivacaine and its efficacy in the symptomatic treatment of burning mouth syndrome [22], tetracaine in polymeric films for the treatment of radiation-induced oral mucositis [20] and dibucaine for relief of pain due to oral erosion [47]. Other classes of drugs with anesthetic properties were also investigated. These include dyclonine for patients with burning mouth and taste alterations [35], mebeverine for oral pain conditions such as traumatic ulcers [39], clove oil for reducing needle pain [34], and capsaicin for evaluating pain models to test the efficacy of topical anesthetics [44,46].

In addition to the evaluation of individual anesthetics, the inclusion of technological strategies to improve the penetration of topical anesthetics was also employed, including Transcutaneous Electrical Nerve Stimulation (TENS) combined with topical application of EMLA^®^ [40,48], and nanocarriers such as liposomes [23,26,27]. These studies aimed to test new active ingredients and to enhance the efficacy of existing anesthetic drugs.

Lidocaine has a long-established history in topical anesthesia, with reports dating back to the 1950s [16,75,76]. In the present bibliometric analysis, this local anesthetic was the most frequently investigated drug in concentrations ranging from 2 to 60% [9,23,38,39,42,44,49,50,51]. It was investigated for its efficacy in reducing needle pain [9,23,38,49,51], reducing pain with argon laser stimulation [42], controlling discomfort from oral ulcers [50], performing scaling and root planing procedures [51], and for comparing different concentrations [42]. Its efficacy was also assessed when encapsulated in drug delivery systems [9,23]. Additionally, lidocaine has been used as a comparison group in tests involving other substances [38,39] and in a study that tested models developed to verify the reliability of experiments related to topical anesthesia with capsaicin [44,46]. These results corroborate the keyword analysis, as the word “lidocaine” appears among the most frequent keywords and is highly interconnected with other terms.

Benzocaine is also one of the most widely used topical anesthetics in Dentistry and was the second most frequently evaluated for its ability to reduce puncture pain in the oral cavity [25,34,38,48,51]. It has been studied for performing scaling and root planing procedures [51] and even for relieving pain due to the use of orthodontic appliances [33].

Prilocaine, commonly used in injectable solutions for infiltration and block techniques (prilocaine 3% associated with felypressin 0.03 IU/mL), began to be extensively studied in topical anesthesia in Dentistry when it was combined in a eutectic mixture with lidocaine, such as the dermatological composition EMLA^®^, in the mid-1990s [77,78,79,80,81]. Subsequently, a prototype of EMLA^®^ for use in the oral cavity was developed, known as Oraqix^®^. Its effectiveness began to be evaluated almost 10 years later [36,45]. In the present study, it was found that this combination of anesthetics was the most studied, employing both formulations, EMLA^®^ and Oraqix^®^ [2,21,23,26,36,37,40,41,43,45].

The majority of studies that evaluated eutectic mixtures aimed to investigate the anesthetic effects, onset, and duration of anesthesia of this formulation during clinical procedures such as scaling and root planing [2,21,36,37,43], reduction in needle puncture pain [23,26,27], reduction in pain during local anesthetic injection [23,26,40], induction of pulp anesthesia [27], duration of anesthesia in soft tissue [27], gum anesthesia [41], and plasma profiles of local anesthetics after application in periodontal pockets [45].

These research focuses may explain why studies on this topic are predominantly published in periodontology journals, with the *Journal of Periodontology* and *Journal of Clinical Periodontology* being the most frequent journals. A recent bibliometric analysis that evaluated papers published in the field of periodontology and implantology showed that the *Journal of Clinical Periodontology*, *Journal of Periodontology*, and *Periodontology 2000* were among the top 10 most cited journals in Dentistry between 2015 and 2019 [62]. Furthermore, the journal *Periodontology 2000* stood out with a large number of citations [61].

In the present review, there is a predominance of publications and citations in the Americas, led by the United States, followed by Europe, Asia, Africa, and Oceania. These results align with a recent bibliometric review that compiled 3300 articles in Dentistry [58]. In that study, the United States also emerged as the leading country in publications. Other studies have also reported that research conducted in the United States is among the most cited [55,63,82]. Furthermore, the majority of funded research originated from the Americas. Notably, only one article from Africa and one from Oceania are among the top 30 most cited, and both lacked funding. The co-authorship analysis conducted in this study highlights that the research groups from Brazil, the United States, England, Japan, Canada, Belgium, and Denmark are the leading contributors in the field.

## 4. Materials and Methods

### 4.1. Search Strategy and Determination of Search Terms

A search strategy with the definition of descriptors and the use of Boolean operators was devised using the advanced search field and the Science Citation Index tool of the Institute for Scientific Information (ISI) in the “Core Collection” section of the Web of Science (WoS-CC) database. WoS-CC was chosen as the primary database for this study to reduce citation bias. The search keys were divided into three main topics of interest: (1) keys related to anesthesia and drugs used; (2) keys related to topical anesthesia; (3) keys related to clinical studies in humans. The search key used was: TS = (anesthesia OR anaesthesia OR anesthetic OR anaesthetic OR anesthetics OR anaesthetics OR cetacaine OR procaine OR EMLA OR lignocaine OR lidocaine OR benzocaine OR xylocaine OR tetracaine OR ropivacaine OR liposomal OR liposome OR dibucaine) AND TS = (topical OR “topical local anesthetics” OR “topical efficacy” OR “topical effect” OR “topical effects” OR “topical application” OR “topical applications” OR needle OR puncture OR injection OR toxicity OR pain) AND TS = (clinical OR “clinical trial” OR “clinical trials” OR human OR humans OR “case report” OR “case reports” OR “case series” OR volunteer OR volunteers OR children OR child OR “randomized clinical trial” OR “randomized clinical trials” OR “randomized” OR RCT OR adult OR adults OR patient OR patients) AND TS = (dentistry OR dental OR “oral medicine” OR “dental surgery” OR periodontal OR periodontology OR odontopediatric OR “pediatric dentistry” OR “paediatric dentistry” OR pedodontic OR pedodontics OR “mini implant” OR “mini implant” OR orthodontics OR “restorative dentistry” OR “dental prothesis” OR endodontology OR implantology OR odontology OR mouth OR tooth OR teeth OR gingiva OR gingival OR facial OR maxilla OR maxillary OR maxillae OR mandible OR mandibular OR “oral mucosa” OR “mouth mucosa” OR intraoral OR “soft tissue” OR “soft tissues” OR pulpal). The search was conducted on 20 October 2023 (citation counts in WoS-CC are updated daily).

**Figure 7:**
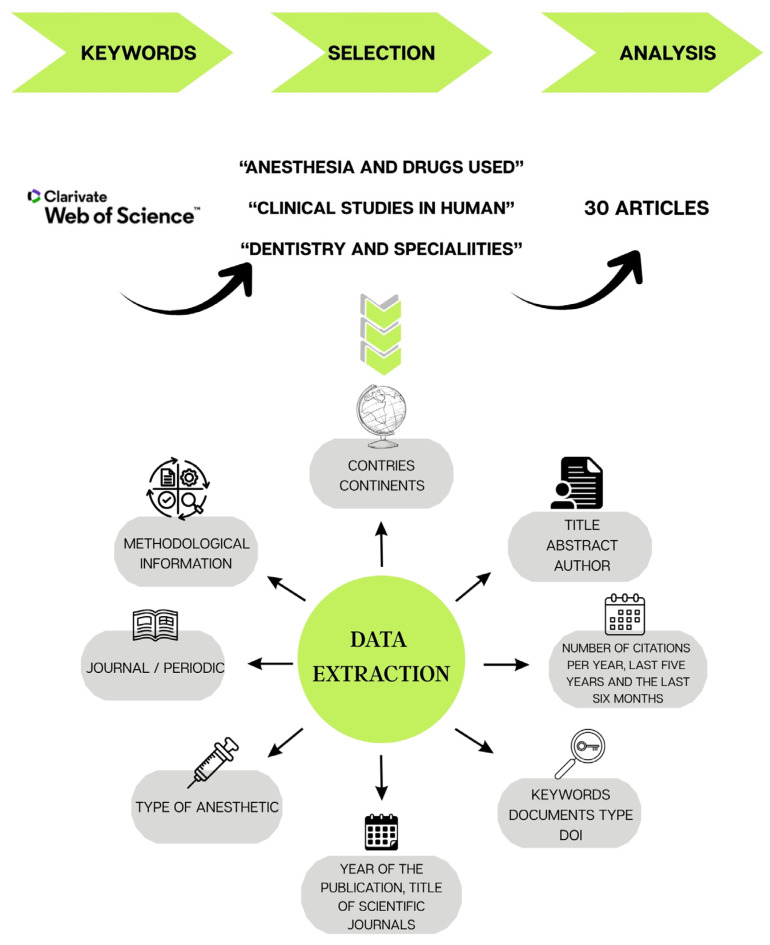
Graphical summary of the methodology used for data extraction.

The selection of articles was carried out by reading the title, abstract, and, when necessary, the full text by a team composed of three evaluators. After the initial selection, all articles were read in full to verify the selection and collect data. Any discrepancies between evaluators were resolved through discussion and consensus. The methodology is summarized graphically in Figure 7. Citation counts were cross-checked with Scopus and Google Scholar for comparison.

### 4.2. Inclusion and Exclusion Criteria

This study included clinical articles on topical anesthesia in dentistry. Documents containing only abstracts, editorial documents, conference articles, letters, and comments, even if related to the proposed theme, were excluded from the analysis in order to avoid duplicate data.

### 4.3. Data Extraction

Data were exported from WoS in TXT and Excel formats. The extracted data included the title, authors, number of citations, average number of citations per year, year of publication, titles of scientific journals, DOI and number of authors. Additional data were extracted from full-text reading and WoS searches, including keywords, abstracts, methodological information, funding, and corresponding author details. Demographic data were inferred from the corresponding authors’ affiliations. The number of citations in Scopus and Google Scholar was also collected and included in the citation counts and citation density as described below. 

In the event of a tie in the number of citations for the studies, they were ranked according to the following criteria: (1) WoS-CC citation density = number of WoS-CC citations/(2022 − year of publication); (2) Number of citations in Scopus; (3) Scopus citation density = number of Scopus citations/(2022 − year of publication).

### 4.4. Data Analysis

Co-authorship networks and keyword analysis were constructed using the VOSviewer software (https://www.vosviewer.com/). Additional graphs, figures, and tables were generated using Canva, Excel, and MapChart (available at mapchart.net).

## 5. Conclusions

Based on the bibliometric review conducted, we observed that studies in the field of topical anesthesia, although relevant, still show a relatively low citation impact. These studies are predominantly concentrated in developed countries. Moreover, there is limited research funding in this area, particularly in Africa and Oceania, where no funded articles were identified among the 30 most cited. Lidocaine remains the most extensively investigated topical anesthetic, whether alone, in combination with other agents, or as a reference compound for efficacy testing of alternative substances. These findings underscore a sustained and growing effort to improve topical anesthetics used in Dentistry, focusing on formulation development, enhanced effectiveness, and reduced toxicity. Benzocaine appears as the second most used topical anesthetic. We also highlight the need for increased financial support and broader global recognition to foster progress in this field, including advances toward needle-free local anesthesia.

## Data Availability

No new data were created. Data sharing is not applicable to this article.
